# The impact of *LEP* gene polymorphisms located at exon 2 (*LEP*-*Hin*fI) and intron 2 (*LEP-Sau*3AI) on growth and reproductive traits in Saanen goats

**DOI:** 10.5194/aab-67-523-2024

**Published:** 2024-10-29

**Authors:** Nursen Senturk, Tugce Necla Selvi, Mustafa Demir, Hakan Ustuner, Hale Samli, Sena Ardicli

**Affiliations:** 1 Department of Genetics, Faculty of Veterinary Medicine, Bursa Uludag University, Bursa, Türkiye; 2 Department of Animal Science, Faculty of Veterinary Medicine, Bursa Uludag University, Bursa, Türkiye; 3 Department of Animal Nutrition and Nutritional Diseases, Faculty of Veterinary Medicine, Bursa Uludag University, Bursa, Türkiye

## Abstract

Leptin (*LEP*), alternatively recognized as the obesity gene, influences food consumption, energy balance, and lipid metabolism. Additionally, it plays a crucial role in energy metabolism, and variations in the *LEP* gene have been studied extensively among various livestock species. These investigations have unveiled correlations with traits such as meat quality, milk yield and composition, and growth characteristics. Nevertheless, the existing knowledge regarding its correlation with growth and reproductive traits in goats is comparatively limited, particularly when juxtaposed with studies of cattle. Hence, the objective of the current study was to investigate the relationship between polymorphisms in the intron 2 (*Sau*3AI) and exon 2 (*Hin*fI) regions of the *LEP* gene and growth and reproductive traits in Saanen goats. The study comprised 185 Saanen goats in total. The PCR-RFLP (polymerase chain reaction restriction fragment length polymorphism) technique was employed to genotype these polymorphisms. Population genetic analysis included the number of effective alleles, heterozygosity, polymorphism information content, and compatibility with the Hardy–Weinberg equilibrium. The general linear model procedure's least-squares methods were used for the statistical analysis. The SNP (single-nucleotide polymorphism) located in exon 2 (*LEP-Hin*fI) was monomorphic. Remarkably, the SNP located in intron 2 (*LEP-Sau*3AI) was associated with birth weight ( p<0.05 ) and average daily weight gain ( p<0.05 ). In this context, the AA genotype demonstrated higher birth weight and daily live weight gain means compared to other genotypes. No significant differences were observed in litter size, total weight gain, or morphometric measurements. The caprine *LEP*-*Sau*3AI polymorphism situated within intron 2 proved influential in traits critical for the profitability and sustainability of goat breeding. The findings of this study provide novel and valuable information for future research on the *LEP* gene in goats.

## 1 Introduction

LEP, a protein hormone secreted by white adipose tissue, influences food intake, body energy homeostasis, and nutrient distribution between tissues, playing a significant role in energy metabolism (Avondo et al., 2019). It plays an important role in regulating and controlling the productive performance of animals (Kumar et al., 2020). LEP is synthesized as a prohormone, which is the early and inactive form of the LEP protein. It consists of 167 amino acids (Hashemi et al., 2011; El-Shorbagy et al., 2022). The *LEP* gene, often referred to as the obesity gene, is located on the fourth chromosome of the goat genome. It is composed of three exons and two introns, with only the second and third exons being translated into protein (Buchanan et al., 2002). The LEP protein, derived from the *LEP* gene, is a 16 kDa protein with diverse physiological effects, including the regulation of body growth, immune function, and reproduction (Singh et al., 2009). In ruminants, the *LEP* gene governs daily food intake, energy expenditure, and metabolic equilibrium, consequently influencing both fat metabolism and muscle development (Barzekar et al., 2009; Houseknecht et al., 1998). Genetic variations in the *LEP* gene are linked to energy balance, milk production, body weight, and fertility traits across various livestock species (Liefers et al., 2002, 2005; Salgado et al., 2022).

In cattle, *LEP* and its receptor (*LEPR*) gene polymorphisms are associated with carcass and meat quality traits (Ardicli et al., 2019a; Kawaguchi et al., 2017), milk yield and composition traits (Banos et al., 2008), and morphological traits (De Matteis et al., 2012). Various studies have investigated polymorphisms of the *LEP* gene located in exon 3 and intron 2 (Liefers et al., 2002; Abousoliman et al., 2020; Singh et al., 2009; Maitra et al., 2014). In cattle, a significant correlation has been noted between the intron 2 variant of the *LEP* gene and production traits (Liefers et al., 2002). Additionally, another study in Holstein–Friesian heifers observed associations with energy balance and first postpartum luteal activity (Liefers et al., 2005). On the other hand, it has been demonstrated that these variants exert significant effects on productivity and reproductive traits in various goat breeds. In a study by Singh et al. (2009), polymorphisms in the exon 2 and intron 2 region of the caprine *LEP* gene were characterized in Indian goat breeds, including Barbari and Jamunapari goats. Through BLAST (Basic Local Alignment Search Tool) analysis of the caprine exon 2 region, it was noted that exon 2 of the *LEP* gene exhibited distinctions in goats compared to other livestock species. Similarly, BLAST analysis of the intron 2 region of the caprine *LEP* gene revealed sequence similarities to cattle and buffalo (Singh et al., 2009).

The variant in exon 2 of the caprine *LEP* gene has been investigated in various goat breeds. However, there is limited information about the frequency and phenotypic effects of this genetic alteration. In Honamli and Hair goats, the *LEP*-*Hin*fI polymorphism in exon 2 was found to be monomorphic (Korkmaz-Ağaoğlu et al., 2019). Furthermore, Surti goats, an indigenous breed of India, showed monomorphism regarding the corresponding LEP marker (Pandya et al., 2020).

Di Gregorio et al. (2014) investigated the impact of the *LEP* intron 1 microsatellite polymorphism on several productivity traits across six goat breeds (Angora, Alpine, Garganica, Girgentana, Maltese, and Red Syrian). The results of their study revealed that, in Red Syrian goats, the two alleles exhibit differing effects on β -hydroxybutyric acid and free thyroxine levels as well as milk somatic cell counts. Additionally, the same microsatellite region showed a tendency to be associated with variations in insulin-like growth factor 1 and triglyceride levels. Darwish et al. (2022) examined the relationship of the *LEP* gene with milk composition in Zaraiby and Aleppo (also known as Damascus, Halep, or Baladi) goats. The study revealed a significant effect on the milk fat content of Aleppo goats. The researchers provided additional evidence supporting the role of *LEP* as an indicator of metabolism and mammary gland health in dairy ruminants. Wang et al. (2015) investigated the relationship between *LEP* genotypes and growth characteristics in Chinese goat breeds. They emphasized that polymorphisms in the caprine *LEP* gene could serve as significant genetic factors affecting growth traits. Furthermore, they suggested that these genetic markers might prove beneficial for upcoming marker-assisted selection (MAS) programs in goat breeding and production. Ziaaldini et al. (2017) showed that genetic variants in intron 2 of the *LEP* gene were significantly associated with weaning weight, body length, body height, and chest girth in Raeini goats. Taken together, evidence has already demonstrated that the *LEP* gene encodes a protein that profoundly influences the regulation of body weight, energy balance, and food intake in mammals. It also plays a pivotal role in numerous biological processes associated with energy metabolism and lipid regulation, and thus *LEP* could be regarded as one of the most efficient biological indicators reflecting body fat levels (Oprzadek and Flisikowski, 2003; Shin and Chung, 2007; Corva et al., 2009; Ardicli et al., 2017, 2019a). While research in cattle genetics has made significant strides, genetic information remains relatively sparse in small ruminants. Despite efforts such as genomic region characterization and variant exploration, studies investigating the genotype–phenotype relationship, particularly in the goat genome, are notably scarce.

Most papers on the subject have focused on production-oriented assessments of goat breeding. However, the reproduction status of small ruminant herds is one of the crucial indices that reflect sustainability and profitability (Ardicli et al., 2021). Hence, molecular genetic evaluations based on growth and reproduction provide pivotal enhancements at the herd level. They are also crucial for a successful goat-breeding program. Despite these observations of *LEP* gene effects, similar studies of goats are lacking, together with current information on the association between *LEP* gene variants and caprine reproduction traits. The Saanen breed, originating from Switzerland, is globally renowned for its high milk production (Korkmaz Ağaoğlu et al., 2012). Its lactation periods vary between 150 and 300 d on average, depending on the country where they are raised, and the lactation milk yield can be between 300 and 2000 kg (Gall, 1980; Rupp et al., 2011; Arnal et al., 2018). Also, the heights at the withers are 80–95 cm in billy goats and 75–85 cm in goats. The live weight varies between 75 and 125 kg for billy goats and between 50 and 80 kg for goats. The hair cover of Saanen goats is short and plain white, with slightly longer hairs on the shoulders, back, and rump (Shelton, 1978; Pesmen and Yardımcı, 2008). Saanen goats are preferred in genetic studies due to their characteristics. To the best of our knowledge, no studies have investigated the correlation between the *LEP*-*Hin*fI and *LEP*-*Sau*3AI genetic markers and the growth and reproduction of this breed. Therefore, this study aimed to examine two SNPs (single-nucleotide polymorphisms) (*LEP*-*Hin*fI and *LEP*-*Sau*3AI) in the *LEP* gene in Saanen goats and to comprehensively assess the impact of these genetic variants on growth and reproduction traits.

## 2 Materials and methods

### 2.1 Animals, management, and sampling

A total of 185 purebred Saanen goats (105 female goats and 80 kids) were used in this study. Ethical approval was received from the Bursa Uludag University Local Ethics Committee for Animal Research (approval no. 2023-06/06). The Saanen herd is located at the Bursa Uludag University Faculty of Veterinary Medicine's Application and Research Farm in the southern Marmara region of Türkiye (40°14′ N, 28°52′ E). Blood samples ( ∼4 mL) from the *vena jugularis* were taken individually into K_3_EDTA vacutainers (Vacutest Kima, SRL, Piove di Sacco, Italy). They were stored at 4 °C and sent to the Bursa Uludag University Faculty of Veterinary Medicine's Laboratory of Genetics.

All the animals were raised under the same feeding and management conditions. The animals were unrelated at least at the parent level. Only healthy goats with complete phenotypic records were included. They were housed indoors and fed concentrated feed in pellet form containing 18 % crude protein, 12 $\mathrm{MJ}\;{\mathrm{kg}}^{-1}$ metabolizable energy, and alfalfa as roughage. During the trial, the goats were provided with free access to water, and 0.50 kg per concentrated feed and roughage was given ad libitum per animal. All the animals were fed twice a day (at 09:00 and 16:00 LT).

### 2.2 Growth and reproduction traits

Phenotypic trait measurement was carried out according to Rashidi et al. (2011), with some modifications. The litter size was considered to be the number of kids born in each parturition. Within 12 h of the kids' birth, the weight of the kids was recorded as their birth weight (BW). From birth until the first breeding weight (FBW) was reached (approximately 12–14 months), the average daily weight gain (ADWG) was determined using the formula ADWG=(FBW-BW)/age of goat at first breeding. Total weight gain refers to the increase in weight from birth to the first breeding of a doe. The growth-related traits, including body length, chest girth, rump height, rump width, and wither height, were determined by the same technicians on the breeding farm. In addition, some morphometric measurements were taken, such as head length, head width, and ear length.

### 2.3 Genomic DNA isolation and genotyping

DNA isolation was performed using a Thermo Scientific GeneJET Genomic DNA purification kit according to the Mammalian Blood Genomic DNA Purification Protocol. Using a spectrophotometer (NanoDrop 2000c), the quantity ( $\mathrm{ng}\;µL^{-1}$ ) and quality (absorbance at 260 / 280 value) of the DNA samples were determined. Pure DNA samples were kept at -80 °C until the genotyping was carried out.

In this study, two SNPs of the *LEP* gene were examined using the PCR-RFLP (polymerase chain reaction restriction fragment length polymorphism) method. The primers used, the PCR conditions, and the corresponding restriction enzymes are shown in Table 1. The DNA amplification reactions were performed using a thermal cycler (Palm Cycler GC1-96, Corbett Research, Sydney, Australia). The PCR amplification was performed in a total volume of 50 µL containing 36 µL of d $H_{2}O$ , 5 µL of 10× buffer, 3 µL of ${\mathrm{MgSO}}_{4}$ , 1 µL of dNTPs (2.5 mM), 2.5 U Taq DNA polymerase (Biomatik, A1003-500U, 5 $U\;µL^{-1}$ ), 1 µL (0.025 µM ) of each primer, and 2 µL of the DNA sample at a concentration of 100 $\mathrm{ng}\;µL^{-1}$ . After amplification, 15 µL of the PCR product with each SNP was digested in 15 units of the corresponding restriction enzyme. These reactions were incubated at 37 °C for 16 h. The digestion products were then electrophoresed in 3 % agarose gel (Sigma-Aldrich, Steinheim, Germany) at 85–90 V for 1 h. The gels were photographed using a gel-imaging system (DNR-Minilumi, DNR Bio-Imaging Systems, Israel).

**Table 1:** *LEP* gene polymorphism characteristics, utilized primers, PCR settings, and restriction enzymes.

SNP	PCR	Primers (5^′^ to 3^′^)	PCR	Restriction	Reference
location	amplification		conditions	enzyme	
	(bp)				
Exon-2 *(LEPHin*f-I)	152	F: 5^′^-TGC AGT CTG TCT CCT CCA AA-3^′^ R: 5^′^-CGA TAA TTG GAT CAC ATT TCT G-3^′^	95 °C 2^′^ (95 °C 1^′^, 55 °C 1^′^, 72 °C 1^′^) 30 cycles, 72 °C 7^′^	*Hin*fI	Singh et al. (2009)
Intron-2 *(LEPSau*3AI)	400	F: 5^′^-TGG AGT GGC TTG TCA TTT CCT TCT-3^′^ R: 5^′^-GTC CCT GCT TCT GGC CAC CTA ACT-3^′^	94 °C 2^′^ (94 °C 1^′^, 55 °C 1^′^, 72 °C 1^′^) 35 cycles, 72 °C 15^′^	*Sau*3AI	Singh et al. (2009)

### 2.4 Statistical analysis

The Court Lab HW calculator was used to test the Hardy–Weinberg equilibrium (HWE). According to the descriptions provided by Nei and Roychoudhury (1974) and Botstein et al. (1980), population genetic indicators such as homozygosity (Ho), heterozygosity (He), effective allele number (Ne), and polymorphism information content (PIC) were calculated. The effects of genotypes on the traits studied were analyzed using the least-squares method as applied in a general linear model (GLM) procedure of Minitab (MINITAB^®^, USA, v17.1.0) according to the following listed statistical models. Birth year, birth season, and litter size were added to the statistical model to evaluate the birth weight of kids as follows:

$$Y_{\text{ijklmn}}=\mu +B_{i}+S_{j}+C_{k}+G_{l}+I_{m}+e_{\text{ijklmn}},$$

where $Y_{\text{ijklmn}}$ is the studied trait, μ is the overall mean, $B_{i}$ is the birth year ( i = 2020–2023), $S_{j}$ is the season ( j is spring, summer, or winter), $C_{k}$ is the litter size ( k is single, twin, or triplet), $G_{l}$ is the *LEP* genotype ( l = AA, AB, or BB), $I_{m}$ are the two-way interactions, and $e_{\text{ijklmn}}$ is the random error.

Concerning the FBW, the regression effect of birth weight was added to the model and tested for significance. Upon identification of significant associations, mean values for each genotype were compared using Tukey's test. The re-parameterized model outlined by Falconer and Mackay (1996) was utilized to compute both additive effects and dominance deviation. In this context, the additive effect was determined as the difference between the means of two homozygous states, divided by 2 ( a ), while the dominance effect was calculated as the deviation of the heterozygote from the mean of the two homozygotes ( d ). The degree of dominance was assessed by the ratio of d to a . If overdominance is present, d exceeds +a or falls below -a .

## 3 Results

### 3.1 Genotypic distribution and population genetic indices

We investigated two SNPs in exon 2 and intron 2 of the caprine *LEP* gene. It was ascertained that the population exhibited monomorphism for the SNP situated in exon 2. Allele and genotype frequencies, population genetic indices (He, Ne, and PIC), and compatibility with the HWE of the polymorphism in the intron 2 (*LEP-Sau*3AI) region are shown in Table 2. The results showed that the selected goat population complied with HWE requirements ( P>0.05 ). The prevalent genotype for *LEP*-*Sau*3AI was observed as AB, constituting 54.37 % of the population. Conversely, the frequencies of the AA and BB genotypes were 36.89 % and 8.74 %, respectively, with the latter being notably less common. Consequently, the allelic frequencies for alleles A and B were significantly disparate, measuring 0.64 and 0.36, respectively.

**Table 2:** Genotype and allele frequencies of the *LEP*-*Sau*3AI marker, population genetic indices, and compatibility with the Hardy–Weinberg equilibrium.

Locus	*LEP*-*Sau*3AI					
Genotypes	AA		AB		BB	
Genotype frequency (%)	36.89		54.37		8.73	
EGF (%)	42		47		13	
Alleles	A			B		
Allele frequency	0.641			0.359		
Ho	0.539762					
He	0.460238					
PIC	0.3543					
Ne	1.8526					
χ2 (HWE)	3.374					
P	0.0662^∗^					

EGF: expected genotype distribution according to HWE; Ho: homozygosity; He: heterozygosity; Ne: effective allele number; PIC: polymorphism information content; χ2 (HWE): Hardy–Weinberg equilibrium χ2 value. ^∗^ P>0.05 : consistent with HWE.

### 3.2 SNP effects on growth and reproduction traits

Subsequent to genotyping, an investigation was undertaken to elucidate the correlation between growth, reproductive traits, and *LEP*-*Sau*3AI. Table 3 presents the least-squares means accompanied by their respective standard errors, along with the significance levels for the *LEP*-*Sau*3AI marker. Notably, this marker exhibited significant associations with two traits: BW ( P=0.024 ) and ADWG ( P=0.005 ). The additive and dominant effects calculated are shown in Table 4. The results showed the presence of overdominance for ADWG and BW.

**Table 3:** The least-square means for the genotype effects of the *LEP*-*Sau*3AI polymorphism on growth and reproduction traits ( n=185 ). P values ​​in bold emphasize that the marker has a significant effect.

Trait	Genotype			Significance ( P value)
	AA	AB	BB	
Litter size	1.83±0.28	1.75±0.27	2.17±0.39	0.370
Total weight gain (kg)	46.70±4.15	46.04±3.97	39.94±5.68	0.320
Body length (cm)	76.57±2.09	77.45±1.24	75.80±2.48	0.816
Chest girth (cm)	14.86±0.77	16.70±0.46	15.60±0.91	0.116
Rump width (cm)	14.83±0.70	15.90±0.38	15.00±0.77	0.318
Rump height (cm)	71.72±0.97	70.86±0.57	70.10±1.15	0.555
Wither height (cm)	69.57±0.89	69.10±0.53	69.30±1.06	0.900
Head length (cm)	22.57±0.49	22.90±0.29	21.60±0.58	0.151
Head width (cm)	13.14±0.56	13.40±0.33	13.60±0.66	0.863
Ear length (cm)	17.00±0.57	16.05±0.34	15.80±0.67	0.298
Average daily weight gain (kg)	0.06±0.01	0.04±0.01	0.04±0.01	**0.005**
Birth weight (kg)	3.05±0.20	2.68±0.21	2.46±0.34	**0.024**

**Table 4:** Additive and dominance effects of markers with substantial associations.

Trait	Additive effect^1^	Dominant effect^2^	Overdominance^3^
Average daily weight gain	0.01	0.01	+
Birth weight	0.295	0.075	+

^1^ The additive effect is estimated as the difference between the means of the two homozygotes, divided by 2 (Falconer and Mackay, 1996). ^2^ The dominance effect is estimated as the nonadditive genetic effects, represented by the deviation of the heterozygote from the mean of the two homozygotes (Falconer and Mackay, 1996). ^3^ The degree of dominance is expressed as d/a . If there is overdominance, d is greater than a or less than a (Falconer and Mackay, 1996).

## 4 Discussion

### 4.1 Genetic variation

In this investigation, we assessed genotypic distribution and population genetic structure by analyzing two polymorphisms located in the intron 2 (*LEP*-*Sau*3AI) and exon 2 (*LEP*-*Hin*fI) regions of the *LEP* gene in female Saanen breed goats. Upon evaluation of the study's findings, it was noted that the *LEP*-*Hin*fI polymorphism exhibited monomorphism within the population. The prevalent genotype in the population was TT, with only two animals possessing the CC genotype, which was consequently excluded from the analysis. As expected, this circumstance has an adverse effect on population genetic indices, which are intricately tied to allele frequency distributions.

HWE serves as a crucial parameter for comprehending the genotypic substructure within a population. Deviations from HWE at specific markers may correlate with population traits. The disequilibrium may arise from inbreeding or indirect selection for these loci resulting from the selection pressure for production traits in many livestock species. However, we observed conformance to HWE for the *LEP*-*Sau*3AI polymorphism. The heterozygous genotype AB exhibits the highest frequency, with the AA genotype closely trailing behind. Conversely, the BB genotype demonstrates a notably lower frequency compared to the other two genotypes (Table 2). In genetic research, population genetic indices are essential for evaluating the structure of a population as defined by genetic variation in specific genes. The levels of the expected He can yield insights into the breeding characteristics of a population; for example, a reduction in He might indicate the occurrence of inbreeding. Furthermore, Ne illustrates the impact of allele frequencies at different loci within populations (Trakovicka et al., 2013). PIC values are commonly employed indices for assessing the degree of polymorphism of a genetic marker. Consequently, the utility of a marker for segregation analysis correlates directly with its polymorphism level. PIC values are the predominant indices used to ascertain the degree of polymorphism in a marker. Accordingly, the utility of a marker for segregation analysis is directly associated with its polymorphism level (Machado et al., 2010; Ardicli et al., 2019b). The calculated He index of 0.4602 and Ne index of 1.8526 suggest a satisfactory level of genetic variability for the investigated *LEP*-*Sau*3AI marker within the analyzed Saanen population. The PIC value was determined to be 0.3543. According to the classification made by Botstein et al. (1980), the PIC values can be shown to be PIC>0.50 (high polymorphism), 0.25<PIC<0.50 (moderate polymorphism), and PIC<0.25 (low polymorphism). Thus, the *LEP*-*Sau*3AI marker examined in this study is moderately informative.

### 4.2 Association analysis

With advancements in molecular genetics, there has been a notable increase in investigations of candidate genes that significantly affect economically valuable traits. The candidate gene approach, employed to pinpoint polymorphisms in genes expected to induce phenotypic diversity based on physiological and biochemical evidence, represents a promising strategy for expediting the enhancement of goat reproductive traits. Recognizing the pleiotropic nature of the *LEP* gene has facilitated its prospective utilization in candidate gene investigations. Studies have documented that genetic variability in the *LEP* gene correlates with diverse traits such as energy balance, milk production, body weight, and fertility across various livestock species (Ardicli et al., 2017, 2019a). While relationship analyses concerning the *LEP* gene in cattle have been conducted to a certain degree, the available data remain notably limited, particularly in goats. Some breeds lack information on certain markers. Moreover, akin to cattle, reproductive and growth or developmental traits may be overlooked, as milk or meat production tends to receive the most attention in small ruminants. It is worth mentioning that the polymorphisms investigated in this study represent the initial examination conducted in Saanen breed goats. To the best of our knowledge, there are no reports delineating the association of these markers with the traits investigated in the Saanen breed. Hence, this study holds significance as an inaugural endeavor in the literature.

In this study, we utilized body characteristics such as body length, rump height, rump width, wither height, head length, head width, chest girth, and ear length, as well as fertility characteristics such as birth weight, litter size, average daily weight gain, and total weight gain for the selection of phenotypic data. Subsequently, we evaluated the relationship between these traits and the *LEP*-*Sau*3AI polymorphism. Our study revealed a significant association between this *LEP* marker and BW ( P<0.05 ) and ADWG ( P<0.01 ). Goats with the AA genotype exhibited a birth weight that was 0.59 kg higher compared to goats with the BB genotype. These animals also exhibit a higher ADWG, accruing 0.02 kg more live weight per day compared to both BB homozygotes and heterozygotes (Table 3). While this disparity may appear minor initially, it should be regarded as significant across the entire population. Consequently, we advocate for an increase in the frequency of the AA genotype within Saanen populations, as this adjustment will engender positive alterations throughout the herd. BW and ADWG, influenced by both genetic and environmental factors, serve as pivotal indicators of breed productivity within commercial goat production systems and are integral to breeding strategies. Furthermore, it is worth considering their relationship with offspring mortality, both at birth and postnatally until weaning. Moreover, assessing the efficacy of preweaning management is essential to achieving the desired survival rate among kids (Awemu et al., 1999; Hailu et al., 2006). These data directly influence the profitability of a goat-breeding enterprise. Hence, the significant associations derived from this study have great importance.

Previous studies have documented the association between the *LEP* gene and body weight and morphometric measurements in ruminants. In a study by Abousoliman et al. (2020) on Barki breed sheep, the rs420693815 locus of the *LEP* gene was identified as having a significant association with ADWG. According to Liefers et al. (2002), the *LEP*-*Sau*3A1 polymorphism located in the intron 2 region of Holstein cattle was linked to milk production and suggested to potentially influence live weight. A study in China involving five breeds examined the relationship between specific SNP regions (g.117T < C, g.1642G < A, g.2883 < A, g.3053 < C, and g.3190G < A) and growth characteristics, finding significant associations. Thus, Wang et al. (2015) concluded that the *LEP* gene emerges as a key candidate gene for growth characteristics. Ziaaldini et al. (2017) investigated the association between the *LEP* gene and body traits in the Raeni Hair goat, revealing significant correlations between weaning weight, body length, body height, and chest girth with the *LEP* polymorphism. De Matteis et al. (2012) assessed the correlation between the *LEP* and *LEPR* gene polymorphisms and milk and morphological traits in Holstein cattle, revealing that the *LEP* gene polymorphism at the g.2003314 G > A locus was linked to rump width and rear legs. Conversely, in our study involving Saanen breed goats, no significant relationship was observed between the *LEP*-*Sau*3AI polymorphism and body measurements (Table 3). Variations in growth and reproductive traits are well-documented among different breeds or populations and even within populations of the same breed under similar environmental conditions. It is crucial to recognize that exploring various combinations of polymorphisms based on genotypic interactions such as epistasis, genetic linkage, and pleiotropy could offer a comprehensive perspective for unraveling the genetic underpinnings of quantitative traits (Ardicli et al., 2021). Moreover, *LEP* is responsible for encoding the hormone bearing the same name secreted by adipose tissue. The concentration of this hormone plays remarkable roles in food intake, energy expenditure, and adipose tissue growth (Buchanan et al., 2002; Banos et al., 2008; Ardicli et al., 2017). However, there is a paucity of information on this topic, indicating the need for further and more extensive investigation into goat genetics.

Reproductive performance is a crucial factor in the sustainability and profitability of ruminant breeding (Vrhel et al., 2024). While the focus on traits like milk yield has led to a decline in reproductive and health traits, especially in dairy cattle, there is now a growing emphasis on considering these traits in selection indices (Ardicli et al., 2019b). This shift is also being observed in small ruminant breeding, with dairy goat breeding seeing a move towards selecting animals with strong reproductive and growth traits. There is a growing interest in expanding selection indices to encompass functional traits such as reproduction and health. This is because solely focusing on production traits can have adverse effects on health and reproductive performance (Miglior et al., 2005). Recent genomic merit evaluations have also reinforced the significance of these traits in ruminant breeding (Ardicli et al., 2024).

One important measure of reproductive performance in goat farming is litter size. Litter size is the number of kids born in each parturition. It serves as a valuable indicator of productivity and sustainability in goat breeding. In our study, we determined the litter size to be 1.83 for the AA genotype, 1.75 for the AB genotype, and 2.17 for the BB genotype regarding the *LEP*-*Sau*3A1 polymorphism. It is evident that the BB genotype exhibited a higher mean litter size. However, this association did not meet the criteria for significance according to Tukey's verification. On the contrary, El-Shorbagy et al. (2022) investigated the impact of the *LEP* gene on production traits and litter size in Egyptian Zaraibi goats, confirming its association with litter size. Limited data exist regarding the correlation between the *LEP*-*Sau*3A1 marker and litter size in goats. Moreover, the breeds examined primarily comprise local breeds specific to particular regions. Therefore, it is imperative to elucidate the impact of these significant genetic markers in more extensively bred goat breeds, such as Saanen. Recent advancements in molecular genetics have facilitated the discovery of new correlations between economically significant traits and genetic markers in animal breeding. The selection of efficient DNA markers linked to these traits, particularly those of high economic value, and the development of innovative genetics-assisted breeding systems have emerged as current priorities in animal breeding and genetics.

## 5 Conclusion

This study focused on the relationship between *LEP* gene polymorphisms and growth traits together with reproductive characteristics in Saanen goats. To the best of our knowledge, this is the first study examining the relationship of the *LEP*-*Sau*3AI region with phenotypic traits in Saanen breed goats. The present study examined the *LEP*-*Sau*3AI polymorphism, which was associated with birth weight and average daily weight gain. The *LEP* gene is one of the pivotal genes that exhibit pleiotropic effects on many crucial biological pathways in mammals. It is also one of the critical candidate genes involved in evaluating economically important traits in livestock species. This study provides evidence for a significant effect of the *LEP*-*Sau*3AI polymorphism on birth weight and average daily weight gain in Saanen goats. It contributes to the missing animal genetic studies in the literature on growth and reproductive characteristics in small ruminants. However, more comprehensive assessments are needed, especially for goats.

## Data Availability

The datasets generated are available from the corresponding author on request.
